# Correlation between Severity of Fetal Growth Restriction and Oxidative Stress in Severe Small-for-Gestational-Age Infants

**DOI:** 10.3390/ijerph182010726

**Published:** 2021-10-13

**Authors:** Mariko Ashina, Takumi Kido, Yuki Kyono, Asumi Yoshida, Shutaro Suga, Ruka Nakasone, Shinya Abe, Kenji Tanimura, Kandai Nozu, Kazumichi Fujioka

**Affiliations:** 1 Department of Pediatrics, Kobe University Graduate School of Medicine, Kobe 6500017, Japan; marikoa@med.kobe-u.ac.jp (M.A.); tkido@med.kobe-u.ac.jp (T.K.); ykyono@med.kobe-u.ac.jp (Y.K.); yasumi@med.kobe-u.ac.jp (A.Y.); sugashu@med.kobe-u.ac.jp (S.S.); rururu_luca@yahoo.co.jp (R.N.); sky.my.kh@gmail.com (S.A.); nozu@med.kobe-u.ac.jp (K.N.); 2 Department of Obstetrics and Gynecology, Kobe University Graduate School of Medicine, Kobe 6500017, Japan; tanimurakenji@gmail.com

**Keywords:** small for gestational age, oxidative stress, fetal growth restriction, z-score

## Abstract

Severe small-for-gestational-age (sSGA) infants exhibit increased mortality and morbidity. Oxidative stress is suggested to be involved in intrauterine growth restriction. This retrospective study aimed to evaluate the oxidative stress level at birth in an sSGA population. Sera of 28 sSGA (sSGA group) and 31 non-sSGA (control group) infants, born at our hospital between March 2017 and March 2020, were evaluated. Oxidative stress (derivative of reactive oxidative metabolites: d-ROM level), biological antioxidant potential (BAP) level, and the ratio of d-ROM/BAP level (oxidative stress index: OSI) were measured. The sSGA group had a significantly lower birth weight (BW), BW z-score, head circumference, and height than the control group (all *p* < 0.05). No significant difference was noted in the BAP level; sSGA infants exhibited a significantly higher d-ROM level than control infants. sSGA infants showed a significantly increased OSI compared with control infants, and the BW z-score was inversely correlated with d-ROM levels and OSI in sSGA infants (R^2^ = 0.300; *p* < 0.01 and R^2^ = 0.319; *p* = 0.02, respectively) but not in controls. In conclusion, sSGA infants, including preterm infants, exhibited higher oxidative stress at birth. The severity of fetal growth restriction was significantly correlated with oxidative stress levels at birth in sSGA infants.

## 1. Introduction

Small-for-gestational-age (SGA) infants are smaller in size than normal for their gestational age infants; these infants usually experience intrauterine growth restriction as fetuses [1]. In addition to increased perinatal mortality and morbidity, SGA infants are at increased risk of developing adult non-communicable diseases as per the developmental origins of health and disease concepts [2,3]. Intriguingly, in recent years, it has been revealed that infants born with severe SGA (sSGA) and birth weight (BW) > two standard deviations (SDs) below the mean BW of newborns [4,5] are associated with more serious complications [1,6,7]. Therefore, an understanding of the pathogenic mechanisms of complications in sSGA is urgently required to establish novel therapeutic strategies for sSGA. To date, several reports have suggested that SGA infants are exposed to prolonged excessive oxidative stress caused by intrauterine malnutrition and hypoxia, although no studies have examined oxidative status in an sSGA population [8,9].

Oxidative stress occurs when reactive oxygen species and free radicals exceed the antioxidant capacity, which can result in oxidative damage [8]. In preterm infants, excessive oxidative stress has been implicated in the pathophysiology of various complications, including bronchopulmonary dysplasia, hypoxic-ischemic encephalopathy, retinopathy of prematurity, periventricular leukomalacia, intraventricular hemorrhage, and necrotizing enterocolitis [8,9,10]. Furthermore, in neonatal intensive care units, several treatments, including oxygen resuscitation, blood transfusions, and phototherapy, and several conditions, including inflammation, infection, and hypermetabolic state, have been suggested as potential causes of excessive oxidative stress in organisms [9,11]. Recently, some studies have found that adverse conditions can cause oxidative stress in multiple tissues and organs [12,13,14]. The effects of oxidative stress on a fetus have been studied, which suggested that increased intrauterine oxidative stress in hypertensive disorders during pregnancy is associated with fetal growth restriction [9]. In a previous study using umbilical cord blood samples taken from term newborns, SGA infants had significantly higher oxidative stress markers than appropriate-for-gestational-age (AGA) infants [9]. Therefore, we hypothesized that oxidative stress might have significant influences on the clinical manifestations of sSGA infants and correlate with the severity of fetal growth restriction. Therefore, we conducted this retrospective study to assess the oxidative status at birth in sSGA infants, including preterm infants.

## 2. Materials and Methods

This retrospective study was approved by the Institutional Review Board of the Kobe University Graduate School of Medicine (approval number 180083). The patients’ parents provided written informed consent for the use of personal medical data. Gestational age was determined based on a dating ultrasound scan during the first trimester. sSGA was defined as a birth weight of <−2 SD for gestational age [5,6]. The serum samples, which were taken at birth and stored at −80 °C for 28 sSGA and 31 non-sSGA control infants who were born at our hospital between March 2017 and March 2020, were used in this study with parental consent. Patients with congenital or chromosomal anomalies were excluded from the study.

Maternal and neonatal data were collected from electronic medical records. Maternal data included maternal age, threatened premature labor (conditions causing subjective symptoms of uterine pain, contraction, bleeding, and/or shortening of uterine cervical length and, therefore, requiring tocolytic agents [15]), premature rupture of membrane (>24 h before delivery [15]), hypertensive disorder of pregnancy (maternal systolic blood pressure >140 mmHg and/or diastolic pressure >90 mmHg during pregnancy [16]), history of maternal smoking during pregnancy, and birth by cesarean section. Neonatal data included gestational age, sex, multiple births, BW, BW z-score, height, head circumference, Apgar scores at 5 min, history of non-reassuring fetal status (NRFS, based on the diagnosis of the obstetricians), and blood gas data (pH, base excess, and lactate and HCO_3_ levels).

Oxidative stress (derivative of reactive oxidative metabolites, d-ROM) and biological antioxidant potential (BAP) were measured using FREE Carrio Duo (WISMERLL, Tokyo, Japan) and then compared between the groups. In addition, the oxidant to antioxidant (d-ROMs/BAP) ratio (oxidative stress index, OSI [17]) was calculated. Regression analysis was performed to linearly compare these parameters and BW z-scores between the sSGA and control infants. Data were expressed as medians (range) or numbers (percentages). The Mann-Whitney U test and Chi-square test were used to compare the sSGA and control data. Differences were considered statistically significant at *p* < 0.05. Analyses were performed using GraphPad Prism version 7.00 (GraphPad Software, La Jolla, CA, USA).

## 3. Results

### 3.1. Patient Characteristics

The clinical characteristics of the infants are presented in Table 1. The sSGA group had a significantly lower BW, BW z-score, head circumference, and height than the control group (all *p* < 0.05). No significant differences were seen in maternal or neonatal blood gas data between the groups.

### 3.2. Oxidative Stress, Antioxidant Capacity, and Oxidant to Antioxidant Ratio in sSGA and Control Infants

**Figure 1:**
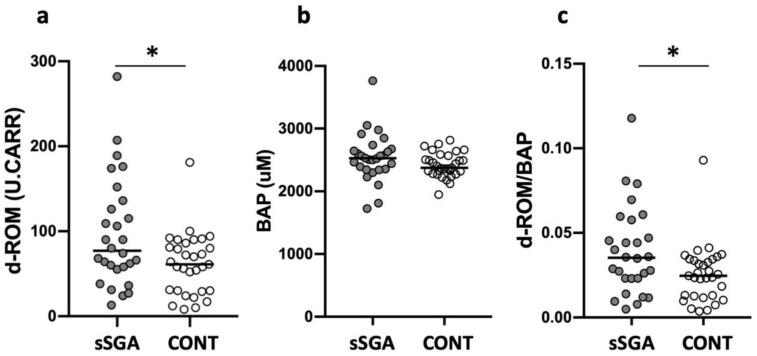
Oxidative stress, antioxidant capacity, and oxidant to antioxidant ratio in sSGA and control infants. White circles represent control infants, and gray circles represent sSGA infants. d-ROM: derivative of reactive oxidative metabolites; BAP: biological antioxidant potential; sSGA: severe small for gestational age; CONT: control. (**a**) d-ROM; (**b**) BAP; (**c**) d-ROM/BAP. * *p* < 0.05.

Although no significant difference was found in BAP (sSGA: 2528 (1725–3765) vs. control: 2376 (1947–2814) µM, *p* = 0.09, Figure 1), sSGA infants exhibited significantly higher d-ROM levels than control infants (sSGA: 77 (13–282) vs. control: 61 (8–181) U.CARR, *p* = 0.03, Figure 1). Additionally, sSGA infants showed a significantly increased OSI compared with control infants (sSGA: 0.035 (0.005–0.118) vs. control: 0.025 (0.004–0.093) µM, *p* =0.03, Figure 1), suggesting a disruption of oxidative balance [17].

### 3.3. Oxidative Stress, Antioxidant Capacity, and Oxidant to Antioxidant Ratio in Preterm and Term sSGA Infants

**Figure 2:**
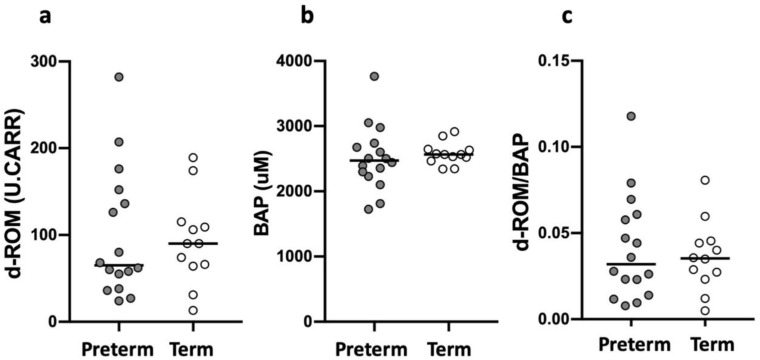
Oxidative stress, antioxidant capacity, and oxidant to antioxidant ratio in preterm and term sSGA infants. White circles represent term infants, and gray circles represent preterm infants. d-ROM: derivative of reactive oxidative metabolites; BAP: biological antioxidant potential. (**a**) d-ROM; (**b**) BAP; (**c**) d-ROM/BAP.

We subsequently divided sSGA infants into preterm (GA: 31 (23–36) weeks; BW: 1117 (284–1792); n = 16) and term (GA: 39 (37–40) weeks; BW: 2155 (1696–2434); *n* = 12) population and compared the level of d-ROM, BAP, and OSI. No significant difference was found in d-ROM (preterm: 65 (24–282) vs. term: 90 (13–189) U.CARR, *p* = 0.72, Figure 2a), BAP (preterm: 2472 (1725–3765) vs. term: 2564 (2341–2916) µM, *p* = 0.37, Figure 2b), and OSI (preterm: 0.032 (0.008–0.118) vs. term: 0.035 (0.005–0.081) µM, *p* > 0.99, Figure 2c).

### 3.4. Correlation among Serum d-ROM, BAP, d-ROM/BAP Level, and BW z-Scores in sSGA and Control Infants

**Figure 3:**
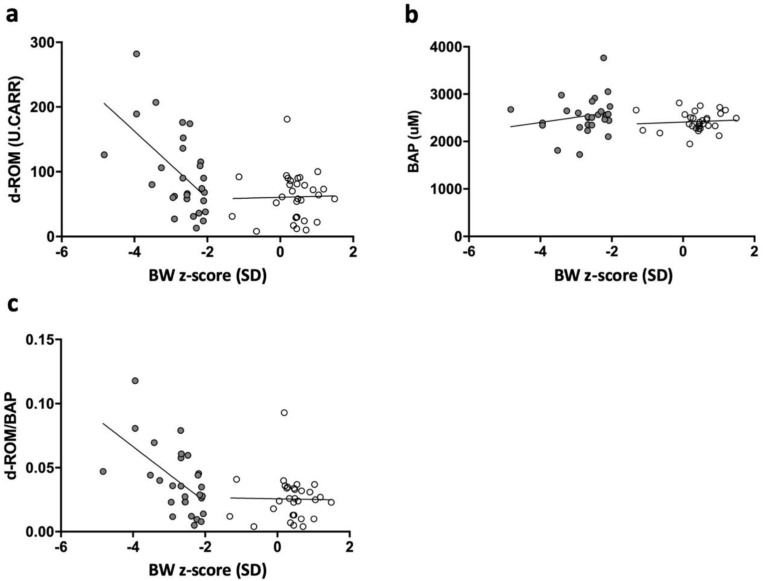
Correlation among serum d-ROM, BAP, d-ROM/BAP level and BW z-scores in sSGA and control infants. Serum d-ROM level and BW z-scores were significantly correlated for sSGA infants (R^2^ = 0.30, *p* < 0.005, Figure 2a) but not for controls (R^2^ = 0.00, *p* = 0.90, Figure 2a). No correlation was found between serum BAP level and BW z-scores in either sSGA (R^2^ = 0.04, *p* = 0.32, Figure 2b) or control infants (R^2^ = 0.01, *p* = 0.65, Figure 2b). Serum d-ROM/BAP level and BW z-scores were significantly correlated for sSGA infants (R^2^ = 0.32, *p* < 0.005, Figure 2c) but not for controls (R^2^ = 0.00, *p* = 0.93, Figure 2c). d-ROM: derivative of reactive oxidative metabolites; BAP: biological antioxidant potential; R^2^: coefficient of determination; sSGA: severe small for gestational age; SD: standard deviation; BW, birth weight. (**a**) d-ROM and BW z-score; (**b**) BAP and BW z-score; (**c**) d-ROM/BAP and BW z-score.

Furthermore, BW z-scores were inversely correlated with the d-ROM level and OSI in sSGA infants (R^2^ = 0.300; *p* < 0.01 and R^2^ = 0.319; *p* = 0.02, respectively), although no correlation was observed in control infants (R^2^ = 0.000; *p* = 0.90 and R^2^ = 0.000; *p* = 0.93, respectively; Figure 3a,c). However, neither sSGA nor control infants exhibited a correlation between the BAP level and BW z-score (R^2^ = 0.038; *p* = 0.32 and R^2^ = 0.007; *p* = 0.65, respectively; Figure 3b).

## 4. Discussion

In this study, we observed that sSGA infants were exposed to significantly higher oxidative stress at birth than control infants, although no significant difference was observed in antioxidant capacity at birth between the groups. Second, the BW z-score was inversely correlated with the d-ROM level and OSI in sSGA infants, but no correlation was observed in control infants.

Gveric-Ahmetasevic et al. measured the serum levels of malondialdehyde (MDA), an oxidative stress marker, in umbilical blood samples at birth and venous blood samples at 3 days after birth in 47 term SGA infants; they reported increased MDA levels in both umbilical arterial and venous blood of SGA infants compared with those of controls, despite no difference in MDA levels in venous blood samples taken at 3 days after birth [9]. Similarly, Gupta et al. measured MDA levels in umbilical venous blood in 20 term SGA infants born from malnourished mothers and found significantly increased MDA levels compared with those of term AGA infants (SGA: 5.33 ± 0.72 vs. AGA: 2.55 ± 0.22 nmol/mL, *p* < 0.01) [8]. It is noteworthy that in our study, the BW z-score was found to be inversely correlated with the d-ROM level and OSI, which might suggest that the severity of growth restriction in sSGA infants is correlated with oxidative stress.

Based on clinical observations, Hussain et al. reported that the BW z-score and the incidence of hypospadias were inversely correlated in an SGA population [18]. Similarly, in a population study examining childhood mortality in SGA infants, sSGA infants (BW <3rd percentile) were reported to have an increased risk of childhood mortality compared with moderate SGA infants (BW within 3rd to 10th percentile) [19]. These clinical findings might reflect the correlation between the severity of fetal growth restriction and the severity of clinical manifestations. Thus, it can be hypothesized that the degree of oxidative stress at birth is related to the postnatal clinical complications of SGA infants.

In this study, the serum level of d-ROM and OSI was significantly higher in sSGA infants than that in control infants; however, no significant difference was noted in the BAP level between the groups. Our results agree with the findings of Watanabe et al., who reported that preeclamptic women with growth-restricted fetuses had significantly increased cord blood levels of d-ROM, but no significant differences in BAP level, compared with those of preeclamptic women without growth-restricted fetuses [20]. In addition, Gupta et al. demonstrated that serum antioxidant marker levels were significantly lower in SGA infants than those in AGA infants, which was in contrast to increased oxidative marker levels (MDA) in SGA infants [8]. Similarly, Hracsko et al. reported increased oxidative stress marker levels (lipid peroxidation) and decreased antioxidant marker levels in the cord blood of term SGA infants compared with those of AGA infants [21]. However, they did not discuss the balance of oxidative stress and antioxidants in each case as this could not be evaluated without measuring the oxidative stress and antioxidant markers in the same case. In this study, we confirmed that sSGA infants were more strongly affected by increased oxidative stress rather than by decreased antioxidant capacity by analyzing the OSI in individual cases. Based on these results, it might be possible to develop new therapeutic strategies for sSGA infants using antioxidants. In particular, pravastatin, a heme oxygenase-1 inducer with antioxidant properties, which has been investigated in clinical trials for the prevention of preeclampsia [22], might be an effective treatment for sSGA infants with oxidative damage.

Furthermore, previous studies regarding SGA and oxidative stress have not included preterm infants, and no reports have examined the level of oxidative stress at birth in preterm SGA infants [8,9]. Previous studies have revealed that preterm infants exhibit higher levels of oxidative stress markers at birth than term infants [10,23]. Several reports have suggested that preterm infants have impaired antioxidant capacity [10]. This study included preterm infants with no difference in patient background between the sSGA and control groups, and we observed that oxidative stress was still higher in the sSGA group than that in the control group. Moreover, when preterm and term infants were compared in our sSGA population, the level of d-ROMs, BAP, and OSI were all similar between the groups. Therefore, we speculated that the increase in oxidative stress at birth in our sSGA population might be due to the effect of growth restriction rather than preterm birth. The relationship between gestational age and oxidative stress dynamics in SGA infants warrants further investigation.

The main limitations of this study were that it was a retrospective study using residual clinical samples with a relatively small sample size. However, the number of patients was similar to that in previous reports [8,9], and we believe that our findings could be important preliminary data for future prospective studies. Additionally, we did not measure maternal oxidative stress and antioxidant capacity, although it has been reported that the mothers of intrauterine growth-restricted infants have significantly higher blood MDA levels than those of AGA infants [24]. Therefore, to clarify the involvement of maternal and placental factors in the pathophysiology of sSGA, it is necessary to measure maternal and neonatal oxidative stress markers simultaneously. Additionally, it would be informative to study the differences between sSGA groups (e.g., sSGA with normal intrauterine Doppler vs. sSGA with abnormal intrauterine Doppler or symmetric sSGA vs. asymmetric sSGA) in future studies. Third, we measured the oxidative stress levels at birth only once; however, it has been reported that preterm infants suffering from perinatal hypoxia have significantly increased oxidative stress even at 7 days of age [23]. Thus, to elucidate the effect of oxidative stress at birth on neonatal clinical characteristics, further prospective studies on postnatal oxidative stress with long-term follow-up are required.

## 5. Conclusions

The severity of fetal growth restriction was significantly correlated with the levels of oxidative stress markers at birth in sSGA infants, suggesting that oxidative stress might play an important role in the perinatal pathophysiology of sSGA infants.

## Figures and Tables

**Table 1:** Clinical characteristics of sSGA and control infants.

	sSGA *n* = 28	CONT *n* = 31	*p* Value
Maternal data			
Maternal age, years	33 (24–40)	34 (22–42)	0.81
Threatened preterm labor	4 (14.3%)	6 (19.4%)	0.60
Premature rupture of membrane	2 (7.1%)	6 (19.4%)	0.17
Hypertensive disorder of pregnancy	6 (21.4%)	6 (19.4%)	0.84
Smoking	1 (3.6%)	1 (3.2%)	0.94
Multiple pregnancy	3 (10.7%)	0 (0%)	0.06
Cesarean section	17 (60.7%)	17 (54.8%)	0.65
Neonatal data			
GA, weeks	36 (23–40)	35 (23–40)	0.88
Extremely preterm infants (<28 weeks)	4 (14.3%)	5 (16.1%)	0.84
Preterm infants (28–36 weeks)	12 (42.9%)	11 (35.5%)	0.56
Term infants (≥37 weeks)	12 (42.9%)	15 (48.4%)	0.65
Male	13 (46.4%)	18 (58.1%)	0.37
BW, g	1728 (284–2434)	2560 (420–3516)	<0.01
BW Z-score, SD	−2.6 (−4.8–−2.1)	0.5 (−1.3–1.5)	<0.0001
Height, cm	40.4 (27.0–47.5)	47.0 (27.0–51.2)	<0.01
Head circumference, cm	29.1 (20.2–40.4)	32.0 (19.6–35.0)	0.02
Asymmetrical SGA *	14 (50%)	-	-
Apgar score at 5 min	9 (1–10)	9 (5–10)	0.07
NRFS	3 (10.7%)	0 (0%)	0.06
Neonatal blood gas data			
pH	7.3 (7.2–7.6)	7.3 (7.2–7.5)	0.57
Lactate	3.6 (1.6–11.5)	3.0 (0.9–6.7)	0.10
Base excess	−1.1 (−12.4–6.1)	0.2 (−6–6.4)	0.24
HCO_3_	24.5 (12.6–29.6)	25.3 (20.1–30.6)	0.12

Data are expressed as medians (range) or numbers (%). * Asymmetrical SGA is defined as a head circumference ≥10th percentile for gestational age. GA: gestational age; BW: birth weight; SD: standard deviation; sSGA: severe small for gestational age; CONT: control; NRFS: non-reassuring fetal status.

## Data Availability

The data presented in this study are available in the article.
